# Modulating DDAH/NOS Pathway to Discover Vasoprotective Insulin Sensitizers

**DOI:** 10.1155/2016/1982096

**Published:** 2015-12-06

**Authors:** Li Lai, Yohannes T. Ghebremariam

**Affiliations:** ^1^Department of Cardiovascular Sciences, Center for Cardiovascular Regeneration, Houston Methodist Research Institute, 6670 Bertner Avenue, Houston, TX 77030, USA; ^2^Department of Cardiothoracic Surgery, Weill Cornell Medical College of Cornell University, New York, NY 10065, USA

## Abstract

Insulin resistance syndrome (IRS) is a configuration of cardiovascular risk factors involved in the development of metabolic disorders including type 2 diabetes mellitus. In addition to diet, age, socioeconomic, and environmental factors, genetic factors that impair insulin signaling are centrally involved in the development and exacerbation of IRS. Genetic and pharmacological studies have demonstrated that the nitric oxide (NO) synthase (NOS) genes are critically involved in the regulation of insulin-mediated glucose disposal. The generation of NO by the NOS enzymes is known to contribute to vascular homeostasis including insulin-mediated skeletal muscle vasodilation and insulin sensitivity. By contrast, excessive inhibition of NOS enzymes by exogenous or endogenous factors is associated with insulin resistance (IR). Asymmetric dimethylarginine (ADMA) is an endogenous molecule that competitively inhibits all the NOS enzymes and contributes to metabolic perturbations including IR. The concentration of ADMA in plasma and tissue is enzymatically regulated by dimethylarginine dimethylaminohydrolase (DDAH), a widely expressed enzyme in the cardiovascular system. In preclinical studies, overexpression of DDAH has been shown to reduce ADMA levels, improve vascular compliance, and increase insulin sensitivity. This review discusses the feasibility of the NOS/DDAH pathway as a novel target to develop vasoprotective insulin sensitizers.

## 1. Introduction

The endothelium represents a monolayer of cells that coat the interior surface of the cardiovascular and lymphatic systems. It is the largest secreting organ in the body weighing up to 1.8 kg and extending to a total surface area greater than 4,000 m^2^ in the vessel wall of adult humans [1]. In the cardiovascular system, the vessel wall is typically composed of extracellular matrix, connective tissue fibers, and smooth muscle and endothelial cells. The number of endothelial cells (ECs) lining the endothelium is believed to be more than one trillion [2, 3]. Physiologically, the ECs are involved in the maintenance of vascular structure and function including formation of physical barrier between blood elements and vessel wall, as well as biological regulation of smooth muscle cell proliferation, adhesion of inflammatory cells to vessel wall, vascular tone, inflammation, thrombosis, and vascular remodeling. These biological effects of the endothelium are mediated by one or more of the molecules that it secretes such as prostacyclin (PGI_2_), thromboxane A_2_ (TXA_2_), thrombomodulin (TM), tissue-type plasminogen activator (tPA), von Willebrand factor (vWF), plasminogen activator inhibitor type 1 (PAI-1), CD39, angiotensin type 2 (AT2), adenosine, endothelin (ET), and nitric oxide (NO) [4]. Among these bioactive molecules, NO is likely the most studied molecule in the pathophysiology of the cardiovascular system including its role in vascular tone regulation, angiogenesis and cell proliferation, inflammatory and immune response, blood clotting, and carbohydrate and lipid metabolism.

## 2. Nitric Oxide Synthesis, Function, and Endogenous Regulation

In the chemical industry, nitric oxide or nitrogen monoxide is known as a byproduct released from heat engines upon refinery of raw materials such as coal, oil, natural gas, and metals. However, mammals are squarely dependent on the biological function of NO ranging from maintenance of blood pressure to immune defense and neurotransmission. Biochemically, NO is synthesized from oxidation of the natural amino acid L-arginine upon catalysis by its three enzymes in the presence of several cosubstrates and cofactors [5]. The first enzyme (NOS I; nNOS) is actively expressed by resident cells of the central nervous system including neurons and nonneuronal cells such as astrocytes, microglial cells, and oligodendrocytes. The main function of NO in the nervous system is its involvement in neurogenesis, neurotransmission, cerebral blood flow, and memory and learning [6]. By contrast, the second NOS isoform (NOS II; iNOS) is mainly expressed by inflammatory cells as an immune defense mechanism including involvement in bacterial killing, cardiovascular inflammation, and neuroinflammation [7]. The endothelial NOS- (NOS III; eNOS-) derived NO is the most active vasodilator and is involved in the regulation of blood pressure, vascular smooth muscle cell proliferation, aggregation of platelets, and adhesion of inflammatory cells to vessel wall [4]. Although it can directly activate downstream targets such as soluble guanylate cyclase (sGC) and calcium-dependent potassium channels to influence vascular function, its half-life is incredibly short measuring only in seconds before its oxidation into nitrites (NO_2_) and nitrates (NO_3_).

Endogenously, the concentration of cellular NO is regulated by several factors including oligomeric structure of the NOS enzymes, concentrations of substrate (L-arginine), cofactors (heme, BH4, FMN, and FAD), cosubstrates (oxygen molecule, NADPH), and intracellular calcium (Ca^2+^). The concentrations of each of these factors are in turn regulated by other factors including enzymes (e.g., arginase regulates arginine), vasoactive molecules (e.g., histamine, bradykinin, and acetylcholine), lipid particles (e.g., oxidized low-density lipoprotein; oxLDL), and reactive oxygen species.

In addition, several studies have demonstrated that the endothelium-derived NOS (eNOS) is endogenously regulated by protein-protein interaction with structural, regulatory, and transport proteins (e.g., with calmodulin, caveolin, actin, tubulin, lipoproteins, soluble guanylate cyclase, and cationic amino acid transporters), posttranslational modification (e.g., phosphorylation,* S*-nitrosylation) triggered by shear stress, hypoxia, inflammatory cytokines (e.g., TNF*α*, IL1*β*), growth factors (e.g., VEGF), and hormones (e.g., estrogen, erythropoietin, and insulin). Moreover, eNOS activity is regulated by its competitive inhibitor asymmetric dimethylarginine (ADMA) [8]. Physiologically, ADMA is synthesized as a result of posttranslational methylation of arginine residues in cellular proteins by a family of enzymes called protein arginine methyltransferases (PRMTs) in the presence of a methyl-group donor* S*-adenosyl methionine (SAM). Once generated and released into the cytosol, ADMA competes with L-arginine for the substrate binding site on the eNOS protein [8]. As a result, the eNOS-bound ADMA inhibits the production of NO and favors the uncoupling of eNOS where highly reactive oxygen (superoxide anion; O_2_^−^) and nitrogen (e.g., peroxynitrite; OONO^−^) radicals are generated instead of vasoactive NO molecules [9]. The concentration of circulating ADMA is normally regulated by urinary excretion and enzymatic metabolism through dimethylarginine dimethylaminohydrolase (DDAH) [8].

In humans, DDAH exists as two isoforms, DDAH1 and DDAH2. Both isoforms have about 60% homology and are differentially distributed across the cytoplasm. Generally, the biodistribution of DDAH1 appears to be colocalized with the neuronal NOS (NOS I) while that of DDAH2 appears to coexpress with eNOS in endothelial and cardiac tissues. Studies have, however, shown that both isoforms can express in various cells and tissue types despite the distribution of the NOSs. For example, in addition to the brain, DDAH1 is known to express in the pancreas, skeletal muscle, heart, liver, and kidneys. Recent genetic [10] and biochemical [11] studies report that DDAH1 is mainly responsible for the enzymatic breakdown of ADMA into citrulline and methylamine. The clearance of ADMA by DDAH is however compromised by several cardiovascular risk factors that impair the expression and/or activity of DDAH [8]. Preclinical and clinical studies have demonstrated that hypercholesterolemia, hypertension, coronary artery disease, renal failure, insulin resistance, and diabetes mellitus are associated with increased production of reactive oxygen species (ROS), decreased expression and/or activity of DDAH, and accumulation of ADMA in cells and tissues. The oxidative stress perpetrated by the cardiovascular risk factors is believed to be the principal cause of impaired DDAH activity due to the sensitivity of the DDAH enzyme to oxidation at a catalytically active site and subsequent loss of enzymatic activity to metabolize ADMA [12].

Higher level of circulating ADMA is a known independent risk factor for major adverse cardiovascular events (MACE) including myocardial infarction (MI) and stroke [13–15]. Elevated plasma ADMA could trigger reduction of endothelium-derived NO through uncoupling of eNOS as described above. In addition, the oxidative stress induced by the cardiovascular risk factors in the vascular wall persistently decreases DDAH activity and reduces NO levels further leading to disturbed hemodynamic compliance. Chronically low level of NO is associated with several morbid vascular disorders and increases the risk of MACE including cardiovascular-related disability and death [16].

By contrast, increased expression of DDAH reduces plasma ADMA and stimulates NO production [8]. Preclinical studies have shown that genetic overexpression of human DDAH1 in mice reduces plasma levels of ADMA by 50% and increases NO by about 2-fold [17]. As a result, the DDAH transgenic animals show optimal vascular compliance including significantly reduced systemic vascular resistance (SVR) and systolic blood pressure (SBP). In addition, cross-breeding of the DDAH1 transgenic animals with the hyperlipidemic ApoE-deficient mice reduces plasma ADMA and decreases the development of atherosclerotic plaque [18].

## 3. NOS/ADMA/DDAH Pathway and Insulin Signaling

Physiologically, endothelium-derived NO (eNO) is known to regulate the transport of insulin and uptake of glucose by several tissues including the endothelium, liver, pancreas, and skeletal muscle [19, 20]. NO enhances flow-mediated vasodilation and likely improves the delivery of nutrients and other chemicals to these target tissues. Conversely, both high glucose and insulin stimulate the transport of L-arginine and increase NO production in vascular endothelial cells [21]. In addition, insulin has been shown to stimulate skeletal muscle blood flow and enhance vasodilation by increasing NO release. The transport of glucose to skeletal muscle is also reported to be regulated by NO. Taken together, studies have demonstrated that insulin-mediated tissue glucose uptake is NO dependent [22]. Thus, understanding the interaction among NO, glucose, and insulin as well as defining the role of the NOS/ADMA/DDAH pathway in this process is essential towards complete characterization and development of effective preventative strategies and novel therapeutic agents for diabetes and its cardiovascular complications, including insulin resistance.

In line with the well-characterized contribution of the NOS/ADMA/DDAH pathway in cardiovascular diseases, the volume of the literature deciphering the precise role of this pathway in the development and progression of insulin resistance, type 2 diabetes, and its complications is growing. Pharmacological and genetic studies indicate that the NOS pathway is necessary in the transport of insulin and glucose to various tissues. Genetic deletion of the eNOS or nNOS genes in mice triggers vascular impairment and insulin resistance [23, 24] likely due to impairment of insulin-stimulated glucose uptake by skeletal muscle and other insulin sensitive tissues. Furthermore, the eNOS knockout mice develop diabetic microvascular complication of advanced diabetic nephropathy in multiple preclinical models of diabetes [25–28].

Similarly, accumulation of ADMA reduces NOS activity and is associated with insulin resistance. As described above, excessive ADMA is often a result of DDAH impairment. The role of dysfunctional DDAH in cardiovascular complications including diabetes and insulin resistance has been studied in several preclinical models [8, 29]. For example, in high-fat-diet-fed type 2 diabetic rats, adipose-tissue-derived DDAH/ADMA is involved in the regulation of insulin sensitivity through modulation of key insulin signaling genes such as insulin receptor substrate 1 (IRS-1) and glucose transporter 4 (GLUT-4) [30]. Homozygous DDAH1 knockout mice are, for example, hypertensive, showing significantly higher ADMA level and decreased NO production [10]. Moreover, targeted deletion of endothelial DDAH1 significantly elevates plasma and tissue ADMA and increases systemic blood pressure [31]. Similarly, homozygous DDAH2 knockout mice are also hypertensive [32]. Several studies have documented that both essential and salt-sensitive hypertension are associated with insulin resistance [33, 34].

By contrast, in DDAH1 overexpression mice, blood glucose and plasma insulin levels after glucose challenge were lower and the insulin resistance index was reduced by 50% indicating enhanced insulin sensitivity in the transgenic animals compared to wild type controls [35]. Likewise, the DDAH2 overexpression mice show enhanced insulin secretion from pancreas despite high-fat diet [36]. Clinically, a number of prospective studies also show that elevated ADMA level is correlated with type 1 or type 2 diabetes [37–42]. Considering all the data from preclinical models and clinical studies, it is evident that the NOS/DDAH pathway is involved in the regulation of insulin and glucose metabolism. In addition, many of these studies indicate that plasma ADMA has a reciprocal relationship with insulin sensitivity. For example, Stühlinger et al. [42] conducted a cross-sectional study evaluating the relationship between plasma ADMA and insulin resistance. The study found that plasma ADMA was higher than normal in insulin resistant subjects. Interestingly, treatment of these subjects with the insulin sensitizing drug rosiglitazone enhanced insulin sensitivity and reduced plasma concentration of ADMA, a correlation that remained significant even after adjusting for risk factors associated with insulin resistance.

## 4. Diabetes, Insulin Resistance, and Cardiovascular Risk

Diabetes mellitus is a complex and multifactorial metabolic disease. Although effective pharmacological approaches and lifestyle-oriented intervention strategies for the prevention and treatment of diabetes have been implemented, the prevalence and incidence of diabetes are still rising at an alarming rate and are particularly worrisome in the developing world. According to the World Health Organization (WHO), the global prevalence of type 2 diabetes, the most common type accounting for 90 to 95% of all cases, in 2014 was about 9% of the adult population. One of the major characteristics of type 2 diabetes is relative insulin deficiency (hypoinsulinemia) or decreased response to insulin stimulation (insulin resistance). In insulin resistant subjects, the body fails to properly respond to endogenous insulin and as a result glucose accumulates within the body instead of being absorbed. This phenomenon overwhelms the insulin-secreting pancreatic beta-cells resulting in gradual loss of the ability to produce sufficient insulin that can dispose the accumulated glucose in the bloodstream. Although insulin resistance alone may not lead to type 2 diabetes, it significantly increases the risk of developing type 2 diabetes. When left uncontrolled, patients with type 2 diabetes are frequently burdened with life-threatening comorbidities including atherosclerosis, hypertension, nephropathy, retinopathy, and neuropathy. Results from several clinical trials including The Diabetes Control and Complications (DDCT), Epidemiology of Diabetes Interventions and Complications (EDIC), and The UK Prospective Diabetes Study (UKPDS) revealed that conventional glycemic control treatment can reduce the risk of cardiovascular events [43].

**Figure 1:**
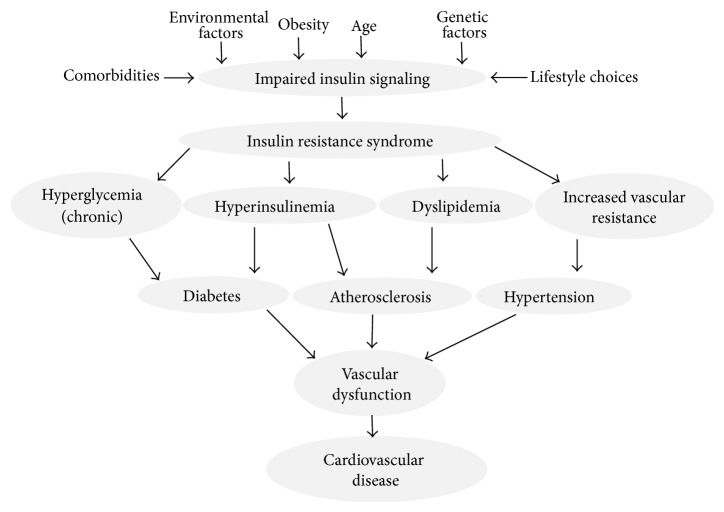
The contribution of impaired insulin signaling to vascular dysfunction and the development of cardiovascular disease.

Besides hyperglycemia, insulin resistance is directly associated with dyslipidemia and hypertension (Figure 1). Insulin resistance plays an important role in the development of dyslipidemia by contributing to an increase in the free fatty acid flux released from insulin resistant fat cells [44]. In addition, hypertensive patients in general and salt-sensitive hypertensive patients in particular are often insulin resistant. Given that the vascular system is a direct target for the action of insulin as described above, all the comorbid complications outlined above heavily contribute to increased cardiovascular risk often seen in diabetic, hypertensive, and/or dyslipidemic patients.

Endothelial dysfunction is an early event in the development of insulin resistance and contributes to many pathological changes in the vasculature including impairment in the contraction and relaxation of blood vessels and inflammatory and coagulation responses, all of which are closely associated with adverse cardiovascular events. More specifically, ECs are directly involved in glucose and insulin uptake. Normally, ECs are stimulated by insulin to produce NO [45]. However, sustained hyperinsulinemia or insulin resistance can lead to progressive decline in endothelium-dependent vasodilation [46, 47], as well as impairment of transcapillary insulin transport to skeletal muscle cells* in vivo* [48].

The interdependence of insulin signaling, glucose metabolism, and endothelial function including the release of endothelium-derived bioactive molecules calls for the development of novel therapeutic strategies to modulate the NOS/ADMA/DDAH pathway to treat insulin resistance syndrome and prevent MACE.

## 5. The Influence of Current Antidiabetes Drugs on Nitric Oxide Pathway

Metformin (Glucophage) is the recommended first-line medication for type 2 diabetes. The mechanism by which this drug suppresses hepatic glucose production and stimulates its tissue uptake is through suppression of mitochondrial respiratory chain complex 1 and activation of adenosine monophosphate- (AMP-) activated protein kinase (AMPK) [49, 50]. Both targeted pathways and the L-arginine-NO pathway are mutually restrained in vascular function and homeostasis [51, 52]. For example, in L-arginine treated ECs, coincubation with an AMPK inhibitor increased glucose accumulation and blunted the increase in NO [51]. Interestingly, the chemical structure of metformin is strikingly similar to that of ADMA and metformin itself is known to reduce ADMA and restore NO levels* in vivo* [53]. In addition, metformin has been shown to protect the liver from inflammatory damage through modulation of the DDAH/ADMA pathway [54] (Table 1).

**Figure 2:**
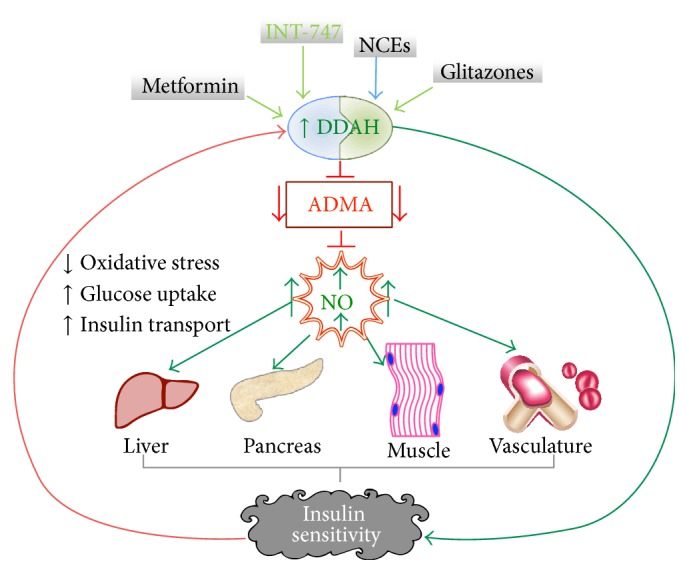
The DDAH/ADMA/NOS pathway and insulin sensitivity. The expression of dimethylarginine dimethylaminohydrolase (DDAH) is upregulated by existing insulin sensitizers such as metformin and pioglitazone or new chemical entities (NCEs). Upregulated DDAH reduces the level of its substrate asymmetric dimethylarginine (ADMA) and increases nitric oxide (NO) production. NO can directly target insulin-responsive tissues to enhance insulin sensitivity. Increased glucose uptake reduces oxidative stress and in turn alleviates DDAH expression.

Thiazolidinediones (TZDs or glitazones) are ligands of the nuclear receptor transcription factor peroxisome proliferator-activated receptor gamma (PPAR gamma) that have recently been developed as insulin sensitizers to treat patients with type 2 diabetes [55, 56]. PPAR gamma is a member of the PPAR family that is mainly expressed in the adipose tissue and is known to masterfully regulate glucose metabolism. Intriguingly, studies have reported that the vasoprotective effect of PPAR gamma ligands is directly correlated with NO regulation. For example, pioglitazone (Actos) has been shown to upregulate DDAH gene expression, reduce circulating levels of ADMA, and enhance NO production [57] (Figure 2). Although this effect could not be reproduced with other TZDs, rosiglitazone (Avandia) has been shown to improve endothelial function (Table 1) in patients with type 2 diabetes [58, 59].

## 6. Modulation of NOS/DDAH Pathway to Enhance Insulin Sensitivity

The effect of NO in insulin signaling is demonstrated in multiple animal models that are genetically modified to manipulate the NO signaling. For example, loss-of-function studies demonstrated that knockdown of eNOS gene reduces NO and causes vascular dysfunction including insulin resistance [24]. By contrast, the NO donor SIN-1 has been shown to rescue the insulin resistance phenotype induced by the NOS inhibitor N(G)-nitro-l-arginine methyl ester (L-NAME) [60–62]. In addition, exogenous supplementation of the NOS substrate L-arginine or the cofactor tetrahydrobiopterin (BH4) is shown to increase insulin sensitivity [63, 64]. These proof-of-principle genetic and pharmacological studies suggest that stimulation of NO production by activating eNOS is an attractive approach to discover and develop novel insulin sensitizers that would also be vasoprotective.

Similarly, increased expression or activity of DDAH reduces circulating levels of ADMA and increases NOS signaling. This strategy is also expected to enhance insulin sensitivity. Recently, we have demonstrated that upregulation of DDAH expression by the bile acid derivative farnesoid X receptor (FXR) agonist INT-747 (Figure 2) improves insulin sensitivity in an animal model of salt-sensitive hypertension and insulin resistance [65]. A clinical trial on the efficacy of INT-747 demonstrated that this compound is able to enhance insulin sensitivity in type 2 diabetics. Meanwhile, we have recently developed a robust high throughput assay to screen for small molecules that modulate DDAH enzymatic activity [66]. Unlike transcriptional DDAH regulators such as the FXR agonists INT-747 and GW4064 that upregulate the gene expression of DDAH (Table 1), our screening strategy is aimed at identifying compounds that modulate DDAH posttranslationally. Using this biochemical assay, we have identified and reported several new chemical entities (NCEs) and existing drugs that directly modulate DDAH activity [66–68]. The effect of these agents on insulin signaling and glucose metabolism remains to be seen. For example, we discovered that the antacid drug, proton pump inhibitors (PPIs), directly inhibit DDAH enzymatic activity and impair endothelium-derived NO [67]. Given that PPIs are among the most widely sold drug worldwide, it might be important, from drug surveillance perspective, to evaluate their effect on glucose metabolism and insulin sensitivity. Meanwhile, when allosteric DDAH activators are developed, it is important to establish their efficacy in glucose metabolism and insulin signaling.

In addition to these pharmacological approaches, dietary supplementation with fruits and green vegetables and other life style choices such as cessation of smoking, body weight management, and regular exercise to reduce oxidative stress and maintain or enhance the NOS/DDAH pathway are possible strategies to prevent and manage cardiovascular disease including diabetes and insulin resistance [69].

## 7. Conclusion

Despite the promising efficacy of the glitazones (TZDs) in improving insulin sensitivity and management of type 2 diabetes, the serious concern over their cardiovascular safety has urged many countries to remove all approved TZDs from the market. In addition, prescription of other antidiabetic drugs such as the sulfonylurea class is increasingly limited due to incidences of associated cardiovascular risk [70]. These unfortunate adversities have intensified the urgency to develop safe and effective insulin sensitizers for diabetic patients. Several targets that are thought to be druggable are being probed for product development. The endothelial NOS/DDAH pathway is presumed to be a viable target to discover and develop novel insulin sensitizers. Preclinical and clinical studies indicate that downregulation of this pathway is associated with insulin resistance and its upregulation is linked to enhanced insulin sensitivity. In addition, upregulation of NOS/DDAH is known to increase the vasoprotective molecule nitric oxide and reduce the cardiovascular risk factor ADMA. Therefore, increased expression and/or activity of endothelial NOS or DDAH using selective NCEs is a novel strategy to develop effective insulin sensitizers that are also expected to protect the vascular wall and reduce major adverse cardiovascular events including stroke and heart attack.

## Figures and Tables

**Table 1:** Insulin sensitizing agents, their mechanisms of action, and associated adverse events.

Insulin sensitizing agent	Mechanism of action	Adverse effect
Metformin	Inhibits mitochondrial respiratory chain complex 1, activates AMPK pathway, decreases hepatic glucose production, stimulates glucose uptake, reduces ADMA, and increases NO	Gastrointestinal discomfort

Troglitazone	PPAR gamma agonist, regulates glucose metabolism, improves endothelial function, and enhances insulin sensitivity	Increases cardiovascular risk, hepatotoxicity

Rosiglitazone	PPAR gamma agonist, regulates glucose metabolism, improves endothelial function, and enhances insulin sensitivity	Increases cardiovascular risk

Pioglitazone	PPAR gamma agonist, regulates glucose metabolism, upregulates DDAH expression, reduces ADMA, increases NO, and enhances insulin sensitivity	Increases cardiovascular risk, bladder cancer

Sulfonylurea	Bind to ATP-sensitive potassium channel on the membrane of insulin secreting cells, increase insulin release, suppress hepatic glucose production, and reduce breakdown and release of fatty acids	Increases cardiovascular risk, severe hypoglycemia

INT-747	FXR agonist, enhances insulin sensitivity, upregulates DDAH expression, and reduces ADMA	Unknown

GW4064	FXR agonist, enhances insulin sensitivity, upregulates DDAH expression, and reduces circulating ADMA	Unknown

AMPK = adenosine monophosphate activated protein kinase; ADMA = asymmetric dimethylarginine; NO = nitric oxide; PPAR = peroxisome proliferator-activated receptor; DDAH = dimethylarginine dimethylaminohydrolase; ATP = adenosine triphosphate; and FXR = farnesoid X receptor.
